# Case Report: Unresectable recurrent nasopharyngeal cancer treated with immuno oncology

**DOI:** 10.3389/fonc.2025.1523481

**Published:** 2025-03-03

**Authors:** Fabiano Flauto, Rosa Maria Di Crescenzo, Vincenzo Damiano

**Affiliations:** ^1^ Department of Clinical Medicine and Surgery, University of Naples Federico II, Naples, Italy; ^2^ Department of Advanced Biomedical Sciences, University of Naples, Naples, Italy

**Keywords:** nasopharyngeal carcinoma, immunotherapy, recurrence, multidisciplinary team, share decision making

## Abstract

**Background:**

Nasopharyngeal carcinoma (NPC) is a rare epithelial malignancy arising from the mucosal lining of the nasopharynx, with significant geographic prevalence in East and Southeast Asia. Despite advances in diagnostic imaging and treatment modalities, over 70% of NPC cases are diagnosed at advanced stages (III/IV), leading to a high risk of recurrence and metastasis. Conventional salvage treatments for recurrent/metastatic NPC, including radiotherapy and chemotherapy, often demonstrate limited efficacy and poor overall survival outcomes. Recently, immunotherapy has emerged as a promising therapeutic approach, modulating the tumor microenvironment and enhancing the immune system’s ability to target cancer cells. This case report describes an unresectable recurrent NPC treated with immunotherapy, underscoring the potential of immune-based therapies in managing advanced NPC.

**Case presentation:**

A 51-year-old male, with a medical history of hypercholesterolemia and a right parietal ganglioglioma, presented with undifferentiated, infiltrating non-keratinizing NPC diagnosed in 2022. Initial MRI and PET/CT scans revealed locally advanced disease, prompting induction chemotherapy followed by chemoradiotherapy. Although the patient experienced several chemotherapy-related complications, follow-up imaging indicated significant tumor reduction. He subsequently underwent concurrent chemoradiotherapy, achieving stable disease. In late 2023, recurrence was identified, and biopsy confirmed EBV-positive NPC. A multidisciplinary team evaluated the case, considering options for re-irradiation and surgical intervention. However, due to the tumor’s location and associated surgical risks, the decision was made to initiate first-line systemic therapy with platinum salts and gemcitabine, followed by immunotherapy with Pembrolizumab. Post-chemotherapy assessments revealed undetectable EBV DNA levels and a complete radiological response. Instrumental reassessment confirmed a complete response, with a negative EBV DNA plasma evaluation. The continued success of systemic therapy and close monitoring remained the focus of the patient’s care.

**Conclusion:**

This case underscores the complexity of managing recurrent NPC and the importance of a multidisciplinary approach. It highlights the evolving role of immunotherapy in treatment strategies, demonstrating its potential to improve outcomes in recurrent/metastatic NPC. Ongoing research is essential to further advance treatment options for this challenging condition. The patient remains under close follow-up, with surgery considered a potential option, given the sustained radiological response.

## Introduction

Nasopharyngeal carcinoma (NPC) is an epithelial malignancy that arises from the mucosal lining of the nasopharynx, often found in the pharyngeal recess. Although considered rare compared to other cancers, NPC is notable for its geographical distribution, with high prevalence in China and in Southeast Asia (1, 2). The etiology of NPC is multifactorial, involving the interaction of Epstein-Barr virus (EBV) infection, environmental factors such as dietary habits and smoking, and genetic susceptibility, including specific high-risk human leukocyte antigen (HLA) alleles. Accumulating studies have shown that tumor-stroma interface withing a tumor is the crucial part of the tumor. Its interface with the stromal components, also termed as the invasive tumor front (ITF), which is commonly defined as the three to six layers of cancer cells at the invasive margin or the disseminated tumor groups between tumor and host tissue. Such evidence suggests a more complex role of tumor microenvironment in NPC pathogenesis, leading to development of wider pathological ecosystem vision (3). According to the WHO, NPC can be categorized into three pathological subtypes: keratinizing squamous, non-keratinizing, and basaloid squamous carcinomas. Non-keratinising nasopharyngeal carcinoma can be divided into differentiated and undifferentiated tumors (4). Among these, the keratinizing subtype accounts for less than 20% of cases worldwide and is particularly rare in endemic regions like southern China. In contrast, non-keratinizing carcinoma constitutes more than 95% of cases in endemic areas and is predominantly associated with EBV infection (5).

Recent advances in diagnostic imaging, the adoption of intensity-modulated radiotherapy (IMRT), and optimized chemotherapy regimens have significantly improved survival rates for NPC patients. Approximately 90% of patients with early-stage disease can achieve a cure with IMRT alone (6). However, due to the anatomical location and the non-specific nature of early symptoms, more than 70% of NPC cases are diagnosed at stages III or IV. Among patients with advanced NPC, 20-30% experience treatment failure, primarily due to recurrence and/or metastasis (R/M). Conventional salvage treatments for R/M-NPC, including radiotherapy, chemotherapy, and surgery, have demonstrated limited efficacy. Current guidelines from the National Comprehensive Cancer Network recommend gemcitabine plus cisplatin as the preferred first-line systemic treatment for R/M-NPC (6). Despite these treatment options, sis remains poor, with a median overall survival (OS) of approximately 20-30 months.

Recent years have seen immunotherapy emerge as a transformative approach in cancer treatment, activating the immune response within the tumor microenvironment and enhancing the ability of immune effector cells to inhibit or eliminate cancer cells. This case report presents an example of unresectable recurrent NPC treated with immunotherapy, emphasizing the potential role of immune-based therapies in the management of R/M-NPC. This case report adheres to the CARE reporting checklist.

## Case presentation

A 51-year-old male, non-smoker, presented with chronic symptomatic right-sided secretory otitis media in early 2022. His medical history includes hypercholesterolemia and a right parietal ganglioglioma, treated with a craniotomy. He remains on chronic therapy with ezetimibe, lacosamide, and bisoprolol. Due to residual post-surgical disease in the right paratrigonal region, he undergoes routine contrast-enhanced brain MRI every six months to monitor for potential recurrence of the brain neoplasm. Upon developing persistent otitis media symptoms that did not resolve with antibiotics, an ENT evaluation was initiated. The persistence of symptoms raised concerns, leading to further diagnostic workup with MRI of the facial region and rhinoendoscopy.

Upon presentation to our facility, the patient underwent an MRI of the facial massif in June 2022, which revealed a notable increase in the size of previously identified heteroplastic tissue on the right postero-lateral nasopharyngeal wall. The mass extended into the nasopharyngeal lumen toward the ipsilateral choana, leading to a reduction in the thickness of the parapharyngeal fat plane. Furthermore, the mass extended to the carotid sheath and infiltrated the middle cranial fossa floor near the right cavernous sinus. Additionally, a right retropharyngeal lymph node, 11 mm in diameter, showed pathological characteristics.

**Figure 1:**
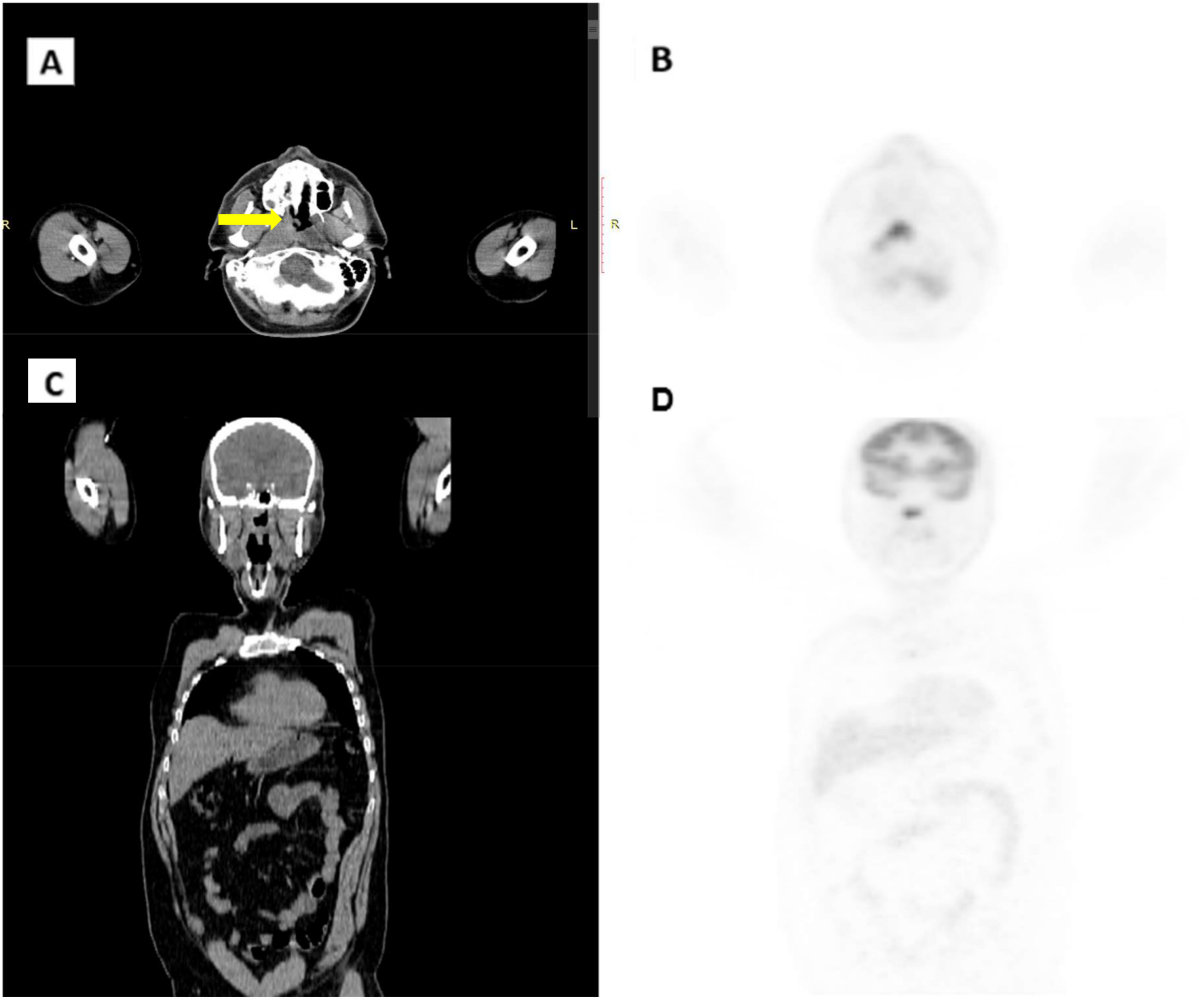
CT/PET scan in June 2022. **(A)** CT axial scan. **(B)** CT coronal scan. **(C)** PET axial scan. **(D)** PET coronal scan. Arrow indicates pathological thickening of the nasopharynx.

**Figure 2:**
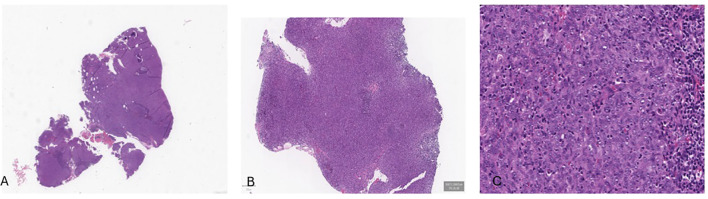
Histological examination revealed a solid tumor **(A-C)**, composed by neoplastic cells with a syncytial appearance, round to oval vesicular nuclei, and large central nucleoli.

A PET/CT scan conducted in late June 2022 confirmed the presence of a solid, heterogeneous oval mass on the right postero-lateral nasopharyngeal wall, measuring approximately 3.2 x 2.2 cm. This lesion caused asymmetry and narrowed the nasopharyngeal air lumen, extending postero-laterally into the carotid space and infero-laterally into the parapharyngeal area. PET imaging showed a high FDG uptake, with a SUV of 18.6, indicating intense metabolic activity consistent with malignancy (**Figure 1**). A nasopharyngeal biopsy performed in June 2022 confirmed the diagnosis of undifferentiated, infiltrating non-keratinizing NPC (**Figure 2**).

The patient, diagnosed with stage III NPC, started induction chemotherapy in July 2022. Treatment involved three cycles of a tri-weekly regimen with Docetaxel 75 mg/m², Cisplatin 75 mg/m², and 5-Fluorouracil 4000 mg/m². Throughout chemotherapy, the patient experienced several adverse effects, including grade 3 neutropenia, grade 2 anemia, grade 2 thrombocytopenia, fatigue, nausea, vomiting, diarrhea, and weight loss of approximately 10 kg.

Following chemotherapy, a contrast-enhanced MRI revealed a notable reduction in the nasopharyngeal mass, with the heteroplastic tissue on the right postero-lateral nasopharyngeal wall reduced to a transverse diameter of approximately 21 mm. The previously identified retropharyngeal lymph node was no longer visible, and there was improvement in the right parapharyngeal space compression.

Concurrently, a PET/CT scan indicated sustained metabolic activity in the residual nasopharyngeal tissue, with no new hypermetabolic areas identified.

Following initial treatment, the patient proceeded with concurrent chemo-radiotherapy, receiving weekly Cisplatin at 40 mg/m² for five cycles, paired with radiotherapy. Radiotherapy was administered via Volumetric Modulated Arc Therapy (VMAT), targeting both the nasopharynx and neck lymph nodes over 33 daily sessions, five days per week. The total prescribed dose was 54 Gy to the nasopharynx and 70 Gy to the neck lymph nodes. During this phase, the patient experienced mild to severe side effects, primarily dysphagia and xerosis of the skin and mucosal surfaces in the oral cavity and pharynx. Due to the occurrence of toxicity, the patient received a suboptimal dose of radiotherapy in the area of the primary tumor.

Post-therapy evaluation in January 2023 revealed some residual thickening of the nasopharyngeal vault without radiologic evidence of active disease, indicating a complete radiologic response.

**Figure 3:**
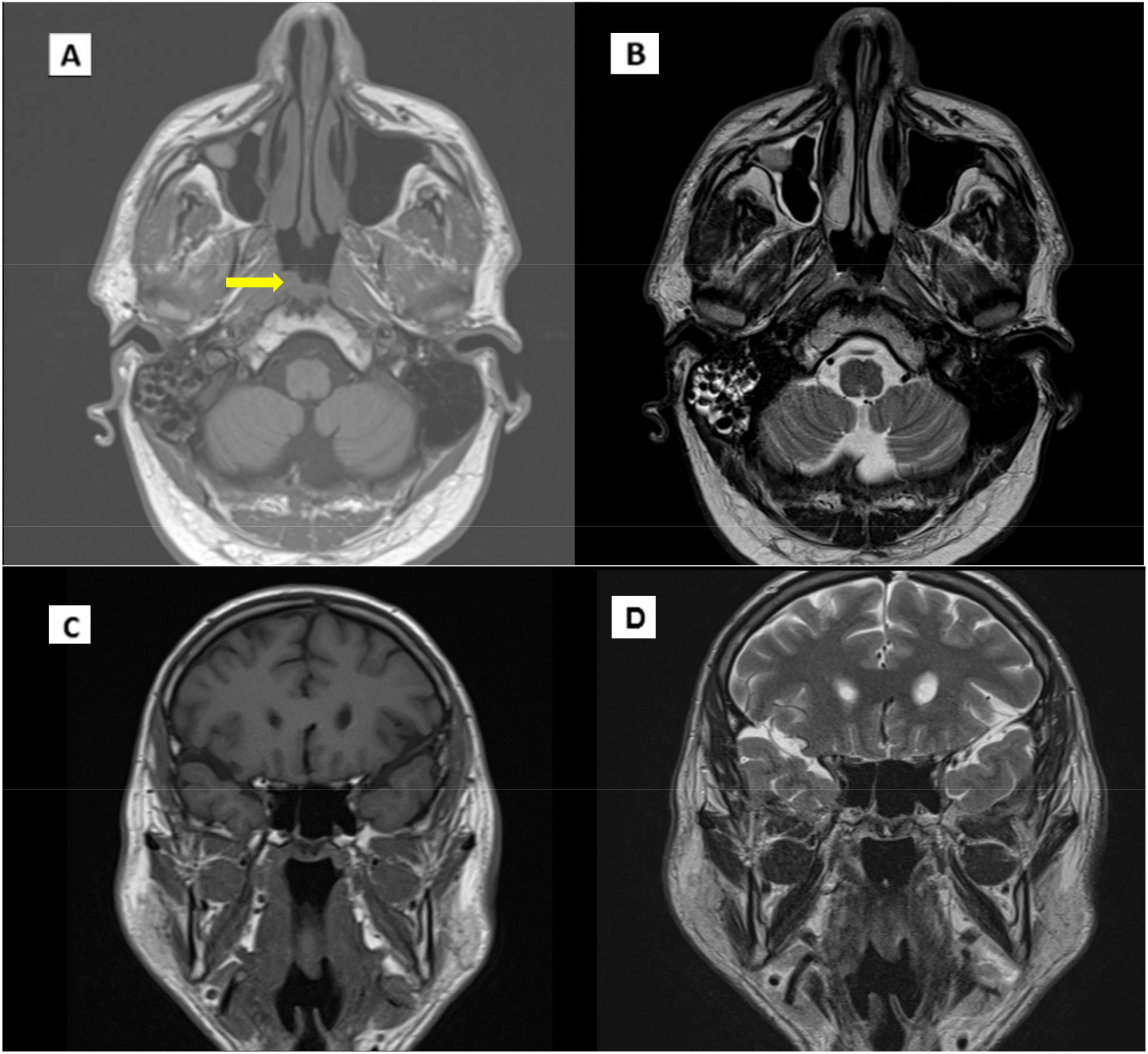
MRI scan in January 2023. **(A)** T1 Coronal scan. **(B)** T1 axial scan. **(C)** T2 coronal scan. **(D)** T2 axial scan. Arrow indicates pathological thickening of the nasopharynx.

Based on these findings, the patient was transitioned to routine clinical and instrumental follow-up (**Figure 3**).

**Figure 4:**
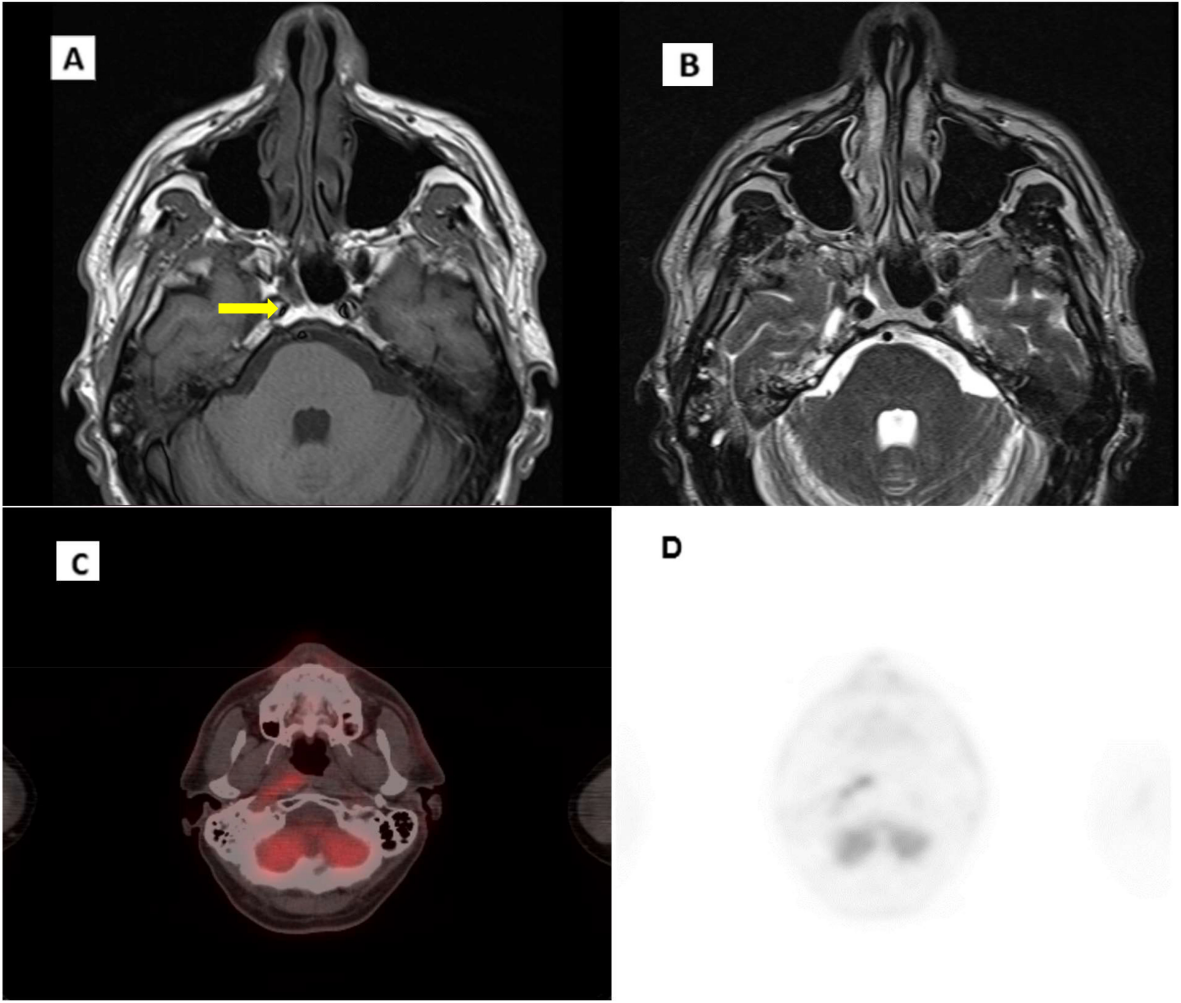
Evidence of disease recurrence. **(A)** T1 MRI axial scan. **(B)** T2 axial scan. **(C)**, **(D)** CT/PET axial scan. Arrow indicates pathological thickening of the nasopharynx.

During follow-up, an MRI of the facial region showed persistent mild thickening of the right postero-lateral wall of the nasopharynx with obliteration of the Rosenmüller fossa. Additionally, a small, 6 mm lymph node was identified in the ipsilateral retropharyngeal region, along with increased mucus retention in both mastoid cavities (**Figure 4**). Given the inconclusive nature of these MRI findings, the images were reviewed comprehensively at our center. In consultation with an ENT specialist, it was determined that continued close monitoring was appropriate, with a planned reassessment in three months.

In October 2023, follow-up imaging with PET/CT, including iodinated contrast, indicated suspected local recurrence. The PET scan showed a tracer uptake in the right posterior nasopharyngeal region, with an SUV max of 6.8, while CT imaging demonstrated mucosal thickening in the corresponding midline and right paramedian area of the nasopharyngeal vault (**Figure 4**). Given these findings, an ENT evaluation was conducted, followed by an endoscopic biopsy. Histopathology confirmed recurrent non-keratinizing nasopharyngeal carcinoma. The biopsy specimen, measuring 8 x 4 mm, displayed syncytial architecture with cells showing vesicular nuclei and prominent nucleoli, consistent with the morphology of nasopharyngeal carcinoma. Immunohistochemical analysis demonstrated strong, diffuse positivity for cytokeratins AE1/AE3 and EBV, while PD-L1 expression had a Combined Positive Score (CPS) between 20 and 25.

Given the disease recurrence, the patient’s case was reviewed by the multidisciplinary oncology team to evaluate rescue therapy options. However, due to the disease’s anatomical complexity, an exploration of advanced radiotherapy and innovative treatment options, including potential inclusion in clinical trials, was undertaken. The team also considered advanced local therapeutic approaches and the feasibility of surgical interventions.

In December 2023, the patient was hospitalized for an ENT-led assessment of endoscopic surgical intervention. During this period, an angiographic study, with a right carotid artery occlusion test, was performed, which unfortunately revealed insufficient compensatory flow from the left carotid artery, precluding the option for immediate surgical resection. Consequently, the team recommended a neurosurgical consult to evaluate the feasibility of a carotid bypass, which was deemed possible. The estimated recovery period following bypass surgery was 40-50 days, after which surgery could be reconsidered. Re-irradiation was deemed unsuitable due to prior high-dose radiation exposure to the nasopharyngeal region and the recent timeline of initial radiotherapy.

During the evaluation period, a new MRI scan of the facial region and neck was performed. The imaging revealed an increase in mucosal thickening at the right nasopharyngeal tubal ostium, with dimensions of 28 x 12 mm on the axial plane, as well as inflammatory changes in the right mastoid and middle ear. The progression of regional disease, albeit mild, underscored the need for timely intervention.

Considering the patient’s ineligibility for radiotherapy due to the increase in tumor mass size, the high surgical risk associated with carotid bypass requirements, the patient was candidate to first-line therapy with platinum salts and gemcitabine. This approach was reached after comprehensive multidisciplinary counseling with the patient.

The treatment commenced in February 2024, utilizing a regimen consisting of Cisplatin at 80 mg/m² and Gemcitabine at 1000 mg/m², administered on days 1 and 8 of a 21-day cycle for four cycels. Throughout the treatment, the patient tolerated the therapy relatively well, maintaining a stable condition despite experiencing mild tinnitus and more pronounced thrombocytopenia.

Post-chemotherapy evaluations yielded encouraging results. An EBV-DNA test in April 2024 indicated that the virus was no longer detectable in the patient’s plasma. A subsequent MRI demonstrated a reduction in the thickening of tissue on the right posterolateral wall of the nasopharynx, suggesting a positive response to treatment. Moreover, a PET/CT scan revealed no areas of abnormal metabolic activity, further indicating that the cancerous tissue was no longer highly active.

Following these favorable assessments, the multidisciplinary team recommended surgical resection of the nasopharyngeal mass at a specialized center. An alternative plan was considered if surgery proved unfeasible; this plan included continuing with up to six cycles of polychemotherapy followed by maintenance therapy with Gemcitabine until disease progression or the onset of unacceptable side effects.

Subsequent MRI scans in May 2024 revealed the presence of a small pericarotid lymph node component, raising concerns about the extent of the disease. Following comprehensive discussions among the multidisciplinary team, the consensus was reached that radical surgery of the nasopharynx, in conjunction with carotid bypass surgery, would offer the best chance for disease control. The indication for the bypass surgery arose from inadequate compensatory blood flow from the healthy carotid artery. Given the identified microscopic disease, this aggressive approach was deemed warranted. If post-surgical histopathological analysis indicated no residual cancer, it would suggest a complete response to therapy, implying a favorable prognosis.

Considering the patient’s clinical status, the extent of the disease, and the necessity for carotid bypass, along with the encouraging radiological and metabolic responses observed following chemotherapy, two treatment pathways were considered:

1. Salvage Surgery: Proceed with surgical intervention on the right nasopharyngeal wall, preceded by carotid bypass, with the goal of complete tumor resection and verification of a full pathological response.
2. Continuation of Systemic Therapy: Maintain systemic therapy while closely monitoring the patient through regular radiological and biochemical assessments, including periodic measurements of EBV DNA levels.

Both options were thoroughly discussed with the patient, emphasizing the risks and potential complications associated with the surgical approach and the carotid bypass procedure. After careful consideration, the patient opted for the second option: to continue systemic therapy with close monitoring through regular follow-ups.

Given the severe thrombocytopenia experienced during polychemotherapy, Pembrolizumab was introduced as part of the ongoing treatment plan. This immunotherapy was administered every three weeks alongside chemotherapy, with the goal of eventually transitioning to maintenance therapy. The patient received the combination of Cisplatin and Pembrolizumab for two cycles between June and July 2024, after which he continued with Pembrolizumab monotherapy.

During the immunotherapy phase, the patient experienced diarrhea, which was effectively managed with antidiarrheal medications and a low dose of prednisone, enabling him to continue treatment with minimal disruption.

**Figure 5:**
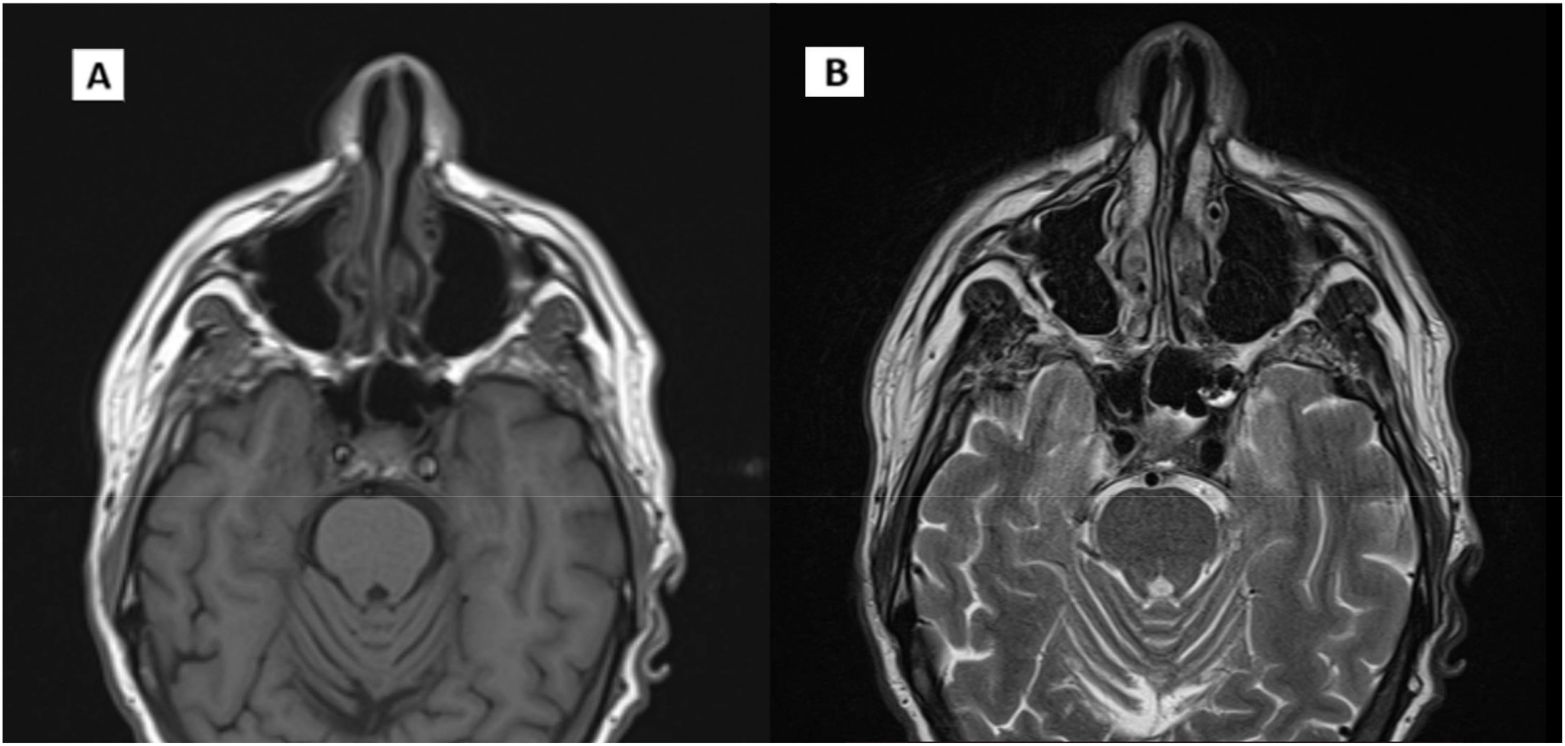
Post IO MRI scans. **(A)** T1 axial scan. **(B)** T2 axial scan.

The patient underwent an instrumental reassessment. These evaluations confirmed a complete radiological response to the disease, and EBV DNA plasma levels returned negative (**Figure 5**). The patient remains committed to the decision to continue systemic therapy with close follow-up, tolerating the treatment well, with only manageable side effects noted.

## Discussion

Managing R/M-NPC presents significant challenges in oncology. NPC is typically treated with radiotherapy, often combined with chemotherapy and targeted therapies, which has improved the 5-year OS rate to 50-64%. However, 10-20% of patients still experience recurrence, driven by factors like previously administered radiation doses, tumor biology, and the recurrence’s location.

For localized recurrences, repeat radiotherapy or surgery is considered. Early recurrences are often radiation-resistant, making surgery the preferred option when feasible. For isolated regional recurrences, neck dissection provides a 5-year OS rate of 41%. Re-irradiation, while potentially effective for low risk cases, carries high toxicity risks, with a reported 33% rate of severe toxicity (7). Chemotherapy is preferred for recurrent disease that is unresectable, unsuitable for re-irradiation, or presents high-risk features (6–8). Salvage surgery for NPC is challenging due to the nasopharynx’s location near critical structures, leading to complications in more than 40% of patients, including facial numbness, trismus, and palatal fistula. Minimally invasive options, like endoscopic nasopharyngectomy developed by Chen et al., have shown comparable results to open surgery while reducing morbidities (9).

Platinum-based multiagent chemotherapy is the standard first-line treatment for R/M-NPC, but options are limited for patients with platinum-refractory disease (10). Although clinical trials for new therapies have stagnated, phase II trials report objective response rates (ORRs) of 54-78% (11). A phase III randomized study demonstrated the superiority of combination therapy with gemcitabine plus cisplatin compared with fluorouracil plus cisplatin in R/M-NPC, with ORR of 64% and 42%, respectively, and median progression-free survival (PFS) of 7.0 months versus 5.6 months (12); however, the exploration of molecular targeted therapy has been hindered by the absence of a definite genetic driver or actionable mutations.

NPC is also linked to EBV infection, especially in WHO class II and III subtypes. EBV infection raises the possibility of immunotherapy, as EBV proteins, such as latent membrane proteins (LMP1, LMP2) and EBV nuclear antigen-1, can elicit a virus-specific immune response. Moreover, expression of LMP1 and interferon-gamma activation in NPC cells can induce PD-L1 expression, which is found in up to 95% of NPC tumors and is associated with poorer outcomes but may predict responses to PD-1 inhibitors.

Given NPC’s high PD-L1 expression and significant lymphocyte infiltration, immunotherapy has shown potential, particularly with EBV-directed vaccinations, adoptive T-cell therapy, and immune checkpoint inhibitors. FDA approvals for immunotherapy agents like nivolumab and pembrolizumab for recurrent or metastatic head and neck squamous cell carcinoma (HNSCC) suggest that these therapies are also applicable to patients with EBV-associated NPC. Exclusion criteria in pivotal trials like KEYNOTE-012 (13), KEYNOTE-040 (14), and KEYNOTE-048 (15) generally included conditions such as active autoimmune diseases, CNS metastases, and infections like HIV and hepatitis, but these therapies remain widely accessible to eligible NPC patients.

Three recent phase III trials, JUPITER-02, CAPTAIN-1st, and RATIONALE-309, have shown significant PFS improvements when adding PD-1 inhibitors to gemcitabine-cisplatin therapy in first-line treatment of R/M-NPC. Toripalimab is a humanized IgG4 kappa monoclonal antibody that binds to PD-1. It blocks PD-L and PD-L2 to PD-1, thereby preventing the inhibition of immune responses via the PD-1 pathway, including anti-tumor immune responses. In the international, double-blind, randomized phase 3 JUPITER-02 trial (16), Mai et al. compared the efficacy of GP chemotherapy combined with toripalimab or placebo as a first-line treatment for R/M-NPC. The results demonstrated that the addition of toripalimab reduced the risk of progression or death by 59%, with a manageable safety profile.

The POLARIS-02 trial reported a 20.5% ORR with toripalimab monotherapy in previously treated patients with R/M-NPC, highlighting the potential of immunotherapy in this context (17).

Similarly, the CAPTAIN-1st trial by Yang et al. showed that camrelizumab combined with GP chemotherapy significantly prolonged PFS compared to chemotherapy alone (9.7 months vs. 6.9 months), with tolerable side effects (18).

The anti-PD-1 monoclonal antibody tislelizumab has been engineered to minimize binding to Fcγ receptors on macrophages and has high affinity and binding specificity for PD-1. The RATIONALE-309 trial randomized treatment-naive R/M NPC patients to tislelizumab or placebo plus chemotherapy (19). At interim analysis, PFS was shown significantly longer with tislelizumab-chemotherapy versus placebo-chemotherapy, regardless of PD-L1 expression.

Pembrolizumab is a highly selective, humanized IgG4/κ monoclonal antibody designed to directly block the PD-1–ligand interaction by binding to PD-1, thereby helping to release the antitumor immune response. The KEYNOTE-028 trial evaluated pembrolizumab across 20 malignancies, including NPC (20).

In KEYNOTE-122, pembrolizumab showed comparable OS to chemotherapy in platinum-pretreated NPC, particularly in patients with higher PD-L1 expression (21). Although OS benefit was not statistically significant, pembrolizumab achieved a 24-month OS rate of 40.2% versus 32.2% for chemotherapy.

In view of the limited predictive value of baseline plasma EBV DNA level, PD-L1 expression level, and Tumor mutational burden (TMB) in R/M-NPC, it is of great interest to find novel biomarkers to predict the efficacy of immunochemotherapy. TMB has been retrospectively correlated with response to immune checkpoint blockade. We prospectively explored the association of high tissue TMB with outcomes in ten tumor-type-specific cohorts from the phase 2 KEYNOTE-158 study, which assessed the anti-PD-1 monoclonal antibody pembrolizumab in patients with selected, previously treated, advanced solid tumors (22).

The phase I/II CheckMate 358 trial assessed nivolumab in virus-associated tumors, including NPC, and demonstrated promising antitumor activity (23). The Mayo Clinic Phase 2 Consortium also studied nivolumab in R/M-NPC, further supporting its potential in virus-associated malignancies (24).

## Conclusion and patient perspective

This case underscores the challenges in managing R/M-NPC, highlighting the necessity of a multidisciplinary approach and the incorporation of advanced therapeutic strategies for these potentially life-threatening malignancies. The importance of multidisciplinary consultation is paramount, as it facilitates shared decision-making between the healthcare team and the patient regarding the treatment pathway. Ongoing research and heightened clinical awareness are vital for improving outcomes in patients with R/M-NPC. Given the tumor’s recurrence and biological aggressiveness, a rigorous follow-up plan involving physical examinations and advanced imaging modalities, such as MRI and PET/CT, was implemented for this patient. The patient chose to begin immunotherapy despite being thoroughly informed about the limited evidence supporting this approach. This is due to the lack of comparable cases documented in the literature. However, the decision was still influenced by the fact that there are ongoing Phase 3 clinical trials for other forms of anti-PD-1 therapy in different populations, which provided some confidence in pursuing this treatment option. Despite the complexities involved, the possibility of revisiting surgical options to achieve a potential cure will be reassessed, especially considering the favorable radiological response observed during the latest imaging evaluations.

## Data Availability

The original contributions presented in the study are included in the article/supplementary material. Further inquiries can be directed to the corresponding author.
